# Competence-Based Trainings for Psychological Treatments – A Transtheoretical Perspective

**DOI:** 10.32872/cpe.13277

**Published:** 2024-04-26

**Authors:** Winfried Rief, Marcel Wilhelm, Gaby Bleichhardt, Bernhard Strauss, Lisbeth Frostholm, Pia von Blanckenburg

**Affiliations:** 1 Division of Clinical Psychology and Psychotherapy, University of Marburg, Marburg, Germany; 2 Institute of Psychosocial Medicine, Psychotherapy and Psychooncology, University Hospital Jena, University of Jena, Jena, Germany; 3 Department of Clinical Medicine, Aarhus University, Aarhus, Denmark; Department of Psychology, University of Trier, Trier, Germany

**Keywords:** psychological treatments, psychotherapy, training, competence, transtheoretical

## Abstract

**Background:**

Although in most countries psychotherapy trainings focus on one treatment orientation, such an approach is associated with systematic shortcomings. The priorities from teaching one theoretical framework should be moved to a more rigorous orientation in science and evidence-based practice, and to the needs of patients, even if strategies of different theoretical approaches need to be combined.

**Method:**

We discuss whether competence-based trainings in psychological treatments offer a better framework to facilitate the progress of psychological treatments to a professional academic discipline with transtheoretical exchange, and we provide an example of a transtheoretical education in the basic competences of psychological treatments. A transtheoretical education program requires an umbrella model for case formulation and a transtheoretical definition of intervention goals.

**Results:**

We provide an adaptation of the traditional model distinguishing vulnerability/resilience, exacerbation, and maintenance of clinical problems for case conceptualization. Dynamic network models offer a further perspective for developing modern, transtheoretical case formulations. Treatment methods should be better classified according to their transtheoretical goals, which offers opportunities to better compare or combine them. We report a case example of how to transform a general competence-based approach in the training of psychological treatments in the academic education system, which found exceptional acceptance from participating students.

**Conclusion:**

Thus, a rigorous competence-based approach to training early clinicians in applying psychological treatments helps to bridge the artificial divide between psychotherapeutic traditions. It also supports the evolution of psychological treatments into an academically robust and highly professional, integrative discipline.

## Highlights

- Effective psychotherapy depends on basic transtheoretical competences of the clinician.
- A competence-based approach helps to overcome barriers caused by the artificial separation of different treatment schools and stimulates better, research-based exchange of findings and intervention effects.
- A case example of training in psychological treatment exemplifies the potential of a transtheoretical training approach and is supported by the exceptional satisfaction ratings of participating students.

In most countries, the training of early career clinicians in providing psychological treatments is highly linked to one treatment tradition (e.g. psychodynamic, cognitive-behavioral therapy CBT) or one newer development in psychotherapy (e.g., Acceptance and Commitment Therapy ACT). Thus, the typical education goal is to become an expert in one of these theoretical frameworks and its practical applications. However, defining psychotherapy as the application of one specific treatment orientation is associated with a series of problems and shortcomings. First, this is in sharp contrast with medical specializations, which have the goal of training upcoming specialists to be able to provide best evidence guideline-oriented treatments for most clinical conditions in the specific field, instead of limiting the training to one specific theory. Such a system, like in medicine, is transparent for cooperating health care providers with other specializations and allows adaptation of training programs according to changes that are based on new evidence, even if other theoretical orientations are necessary to understand and use the new guidelines. As long as psychotherapy is defined through separated theoretical orientations, the transtheoretical stimulation and inspiration of treatment experiences are hampered, and a consequent transition of scientific evidence to clinical application (and back) is limited. Even for the blockbuster of scientific evaluation in psychological treatments, CBT, an exclusive perspective on its own concepts hinders dynamic progress that would allow for benefits from other experiences outside its own theoretical world.

Defining psychological treatments as a family of non-linked theoretical orientations is also in opposition to research results that highlight that successful treatments and successful therapists have shared features that are not limited to one single theory (Norcross & Lambert, 2019). Common factors explain major parts of the variance of outcome (Wampold et al., 2017). Further, there seems to be a benefit if therapists have options to switch to interventions from other theoretical backgrounds, or, as Fonagy has pointed out: “Recent studies indeed suggest that adherence flexibility ([...] using interventions from other treatment approaches and modalities) may be associated with superior outcomes” (p. 270, Fonagy & Luyten, 2019). However, the typical trainings of early career clinicians do not sufficiently address this full potential of scientifically based knowledge about the delivery of effective treatments, and improved education in transtheoretical competences can provide a pathway to more successful treatments in clinical practice.

The lack of a common language for psychological treatments further hinders fruitful exchanges between representatives of different treatment approaches. Overcoming these restrictions offers new potential for improving training for psychological intervention and for shaping the personal competence profiles of upcoming psychotherapists. In addition, this leads to more transparency in what patients can expect from an expert providing psychological treatments. It seems barely acceptable that patients have to inform themselves before they choose psychological treatments about whether the treatment provider has a good training, is able to offer guideline-oriented treatments, or has some specialization that does not fit the patient`s problem. Like in other fields of healthcare specialization, patients have a right to expect that experts providing treatments for mental health should be qualified to address most clinical problems in this field with the best evidence intervention.

## Competence-Based Training as a New Framework for Education in Psychological Treatments

What are the competencies that patients can expect if they search for an expert offering psychological treatments? It is surprising that many groups trying to define basic competences for clinical psychologists came up with a list of general competences that are not linked to one specific orientation but rather take into consideration the clinical needs and experiences with patients suffering from mental and behavioral disorders. The University College of London has done impressive work in defining competence profiles, some of them being linked to clinical conditions (such as psychosis), and some of them being linked to providing specific treatment approaches (UCL, 2020). The problem-specific definitions of competences needed to professionally treat this condition summarize a long list of general factors before defining the specific competences that are necessary to provide a specific treatment approach (see example for persistent physical symptoms; Supplementary Materials, Figure S1). The European Association of Clinical Psychology and Psychological Treatments (EACLIPT) also provided a list of general competences of professional clinical psychologists (EACLIPT Task Force on “Competences of Clinical Psychologists”, 2019) that was the result of a discussion group with members representing different treatment orientations (for an excerpt, see Supplementary Materials, Table S1).

We can use such competence profiles as a starting point for the systematic development of a self-learning system (Rief, 2021): Trainings for therapists can better focus on these competences, and the consequences for patients and other involved people can be evaluated. The results of this evaluation can be fed back into the competence list, leading to refinements and changes. Therefore, comparable to the English “talking therapies” program (Clark, 2018), such a living system can lead to the detection of weaknesses in current mental health care, and the ability to respond with methods to improve the system.

A competence-based approach typically indicates that people providing professional psychological treatments need to have general academic knowledge that is relevant for understanding the clinical condition (e.g., from basic psychology or neuroscience), mainly to have a basic understanding of evidence-based change processes during treatments, and they need the personal competences to apply this knowledge in the current patient-clinician-interaction.

However, moving from a theory-specific training of clinicians to a transtheoretical, competence-based training has some requirements. First, we need a general, transtheoretical framework for case conceptualization. Second, we need some agreement about the necessary competences and how interventions from different treatment orientations can contribute to the training of these competences.

## Transtheoretical Case Conceptualization

**Figure 1:**
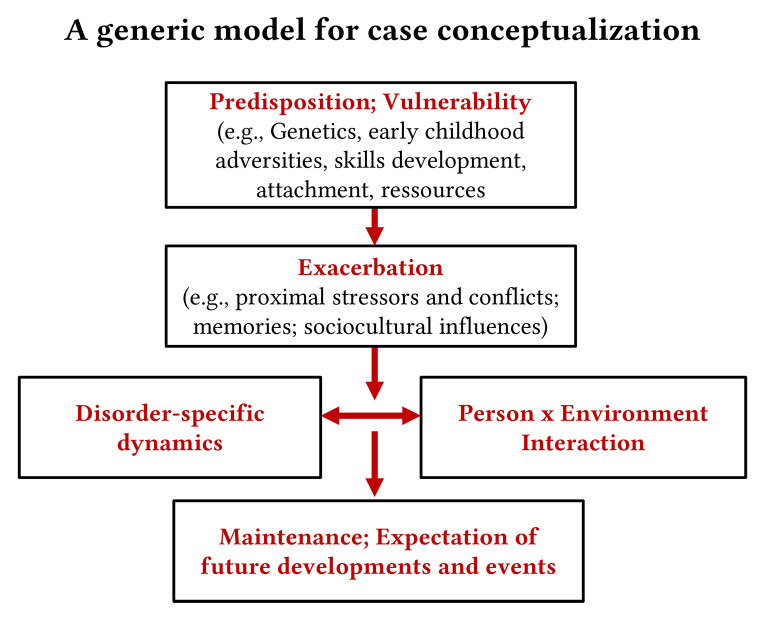
Transtheoretical Case Conceptualization (Adapted From Rief & Strauß, 2018)

Transtheoretical case conceptualizations are necessary to provide a framework for understanding mental disorders and to identify foci for personalized treatment decisions. One of the oldest transtheoretical concepts for case conceptualization is the diathesis-stress model which distinguishes vulnerability/resilience factors (distal factors) from factors that led to symptom exacerbation (proximal factors), while symptom persistence and chronicity is closer linked to maintaining factors. This model has been modified by Rief and Strauß (2018; Figure 1) to better integrate person-environment interactions, the self-perpetuating capacity of mental disorders (disorder-specific dynamics), and the specific role of patient expectations as a maintaining factor (Rief & Glombiewski, 2017). Problems in social interaction are a scientifically proven risk factor for the development of mental disorders (starting from attachment experiences in early life; see predisposition box in Figure 1), but social interaction problems can also develop or intensify after the establishment of mental disorders, thereby contributing to maintaining mechanisms (see person x environment interaction and maintenance boxes in Figure 1).

The model presented in Figure 1 offers a guidance for designing training programs for psychotherapists: they need to acquire competences to address problems of every single box, and they have to decide which box requires most attention according to the individual case conceptualization. It also highlights that mental disorders can have their own intrinsic dynamics, and typically this needs to be addressed directly. Although the classification of mental disorders is under discussion (Rief et al., 2023), therapists need specific competencies to address different mental disorders (disorder-specific dynamics).

**Figure 2:**
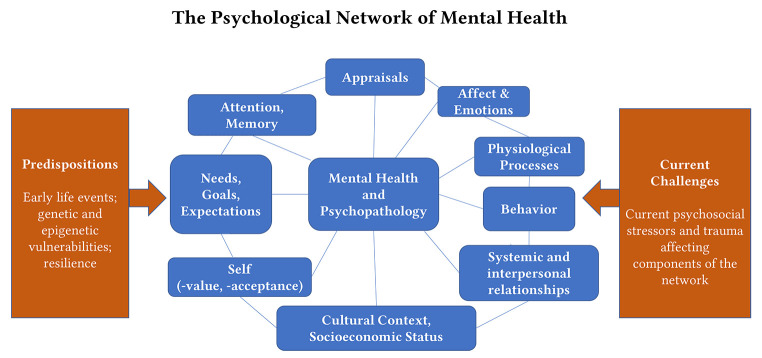
A Network Approach as a Common Framework for Transtheoretical Treatments (Lutz & Rief, 2022)

While models such as the one in Figure 1 are still mainly static, dynamic network models for understanding mental disorders have been published to overcome the limitations of our traditional case conceptualizations (see Figure 2). These models identify “nodes” with high centrality to understand the clinical condition, and these nodes can vary from patient to patient. Therefore, network models do not only overcome the gap between group-oriented (nomothetic) concepts and person-oriented (idiographic) conceptualizations, but they also explain why sometimes different treatments can lead to the same results, while in other cases the same treatments can lead to very different results (depending on the network status of the patient). Further, such a network approach rejects the illusion of separated clinical disorders, takes it as given that symptoms and clinical problems can be highly interlinked and that mental disorders do not represent isolated entities. While applying network models to clinical decisions and treatments is just at its beginning, first attempts show highly promising results, open the view to a more transtheoretical understanding, and enable us to define new pathways for treatment planning (Betz et al., 2020; Fried & Robinaugh, 2020). Of note, clinical information can be integrated into data-driven developments of individual network models (Burger et al., 2022; Scholten et al., 2022).

To ensure a comprehensive competence-based training, clinicians are trained to integrate both the traditional diathesis-stress model and the evolving network models, recognizing the unique contributions and insights each brings to understanding and treating mental disorders. Trainings of clinicians should qualify to address every single node (see boxes in blue, Figure 2) if it is considered a critical part of the network.

## Transtheoretical Categorization of Psychological Interventions

Instead of describing treatment goals with the words of one specific treatment approach, we recommend categorizing treatment techniques according to more general aims using the basic concepts of psychology and neuroscience. This helps to bundle treatments from different treatment theories, thereby indicating the potential for comparing, stimulating and evaluating similar approaches and accelerating their developments to better achieve the common goal. The selection of these treatment goals is grounded in a transtheoretical framework, emphasizing the importance of versatile skills that transcend specific theoretical orientations, thereby ensuring clinicians are well-equipped to address the complex needs of their patients. Beyond integrating academic knowledge into clinical work and considering disorder-specific recommendations, clinicians providing professional psychological treatments should be trained to develop the competencies according to the following treatment goals.

### Establishing a Therapeutic Relationship

It is not only common sense that the quality of the therapeutic relationship is able to predict treatment outcome, but it is also an everyday experience in the context of clinical encounters that the trustworthiness of the clinician is a major predictor of a patient’s behavior and whether a patient accepts and complies with a therapist´s recommendation. While this is not part of the case conceptualizations in Figure 1 and Figure 2, nearly all general models of psychotherapy emphasize the role of a therapeutic relationship as a precondition for treatment success, although much of its evidence goes back to correlational analysis (Grawe, 2004; Norcross & Lambert, 2019; Wampold & Imel, 2015). There is ambiguity regarding how to define the relevant features of therapeutic relationship. In social psychology, one of the most prominent concepts on social perception is the model of Fiske and others (Fiske et al., 2007; Fiske et al., 2002), summarizing that the major features of social perception can be grouped into the two factors of warmth and competence. In one of the few studies using an experimental approach to investigate the role of therapeutic relationships, we were able to show that both warmth and competence determine whether participants make use of new information provided by a therapist (Seewald & Rief, 2023). This does not only determine explicit change processes but also implicit attitudes (Seewald et al., 2023). This means that the new experiences triggered during treatment sessions can only be integrated if patients consider the therapist as someone with warmth (empathy, perspective taking, non-aggressive) and competence (e.g., well-trained, providing convincing explanations, structuring treatment sessions). A first step in training early career clinicians should be how to establish a relationship with a patient that leads to the patient’s perception of a therapist as being warm and competent.

### Consideration of Patient´s Goals and Values

In the past, motivation for psychotherapy and motivation for change have been typically considered as preconditions for treatment. This has substantially changed over the last two decades, and working with motivation is considered a part of the psychological treatment process, in particular if the motivation for treatment and for change is fragile, ambiguous, or varies because of conflicting needs. Therapists need the competence to reflect the patient´s motivation and needs, and to consider the patient´s life goals during the treatment process, to finally arrive at shared treatment goals to select intervention techniques that are in accordance with the patient’s general values. Acceptance and commitment therapy (ACT; Hayes et al., 2006) has reinvented the consideration of existential life goals to establish commitment as part of the treatment process, but the tradition of working with patient´s life goals and values is much older (e.g., Frankl, 1955). Psychodynamic treatments often focus on the conflict between different motives of patients. But also Roger´s non directive intervention aims to clarify patient´s needs, help to find solutions in conflicts, and to increase the motivation to follow them. Thus, people offering professional psychological treatments need the competence to analyze, reflect and work with the patient´s motives, taking into consideration general life goals and values of patients, to clarify different aspects of conflicts, and to improve patient´s motivation for change. Motivational interviewing is just one of the examples of how to directly focus on aspects of motivation (Miller & Rollnick, 2002); originally developed for people with addiction problems, it can be used for nearly all decision problems, as a tool to improve motivation for change in particular with patients with stable, dysfunctional states (e.g., a patient with anorexia suffering from chronic underweight; long-year persisting depressive states; dysfunctional aggressive and impulsive behavior). This strategy can be easily combined with other treatment techniques.

### Improving Tolerance for Unpleasant Sensations and Feelings

Emotion regulation refers to the process of understanding, managing, and effectively coping with feelings and sensations. It involves developing skills to identify and respond to emotions in a healthy manner. This can include recognizing triggers, understanding the intra- and interpersonal context of feelings, and implementing strategies to manage intense feelings. The rise of concepts on emotion regulation and their relevance in psychological treatments also brought another insight into the field that has its roots in the Buddhist wisdom “Living is suffering”. Every person needs competence in tolerating aversive states and not to change strategies because of single unpleasant disruptions. People suffering from chronic aversive states (e.g., chronic pain) need to develop acceptance strategies, if they want to improve their quality of life. Therefore, clinicians should be able to support patients how to better tolerate unpleasant feelings.

### Improving Skills

The counterpart of accepting aversive situations and memories is trying to change them. This often requires the improvement of skills, and improving skills is a major component of nearly all treatments. The history of training how to improve communication skills started before behavior therapy was officially introduced (Salter, 1949), and psychodynamic treatments wanted to overcome “structural deficits” (e.g., deficits in emotion regulation, communication of needs, self-concepts) by working with the patient´s psychological skills during the therapeutic encounter. Other skills were added to the portfolio of skills improvements in psychological treatments: Improving problem solving skills, relaxation skills, emotion regulation, and mentalization competence (reflection of motives and emotions of others and self). These interventions focusing on improving skills have some specific characteristics in common. They typically follow a step by step approach, trying to induce some smaller successful changes as soon as possible, before aiming for broader goals. They typically follow a communicable rationale and are rooted in the principles of learning.

### Exposing to New and Feared Situations

Although exposure is often defined as a pure CBT intervention, the overall goal is broader: how to expose a patient to a new situation, a feared situation, or an aversive inner stressful experience if this is necessary to achieve the treatment goals? With such a definition, it is obvious that every psychological treatment will arrive at such a point because either implicitly or explicitly most patients have to face the fact that exposure is a prerequisite for change. Most treatment frameworks require exposure to new situations and/or behaviors (Foa & McLean, 2016). Further, there are few psychological interventions with as much scientific evidence and scientifically based principles as exposure. Therefore, knowing about the basic principles of exposure interventions and being able to motivate and guide patients through such a process is a basic requirement for all therapists.

### Working With the Therapeutic Relationship as an Example of Interactions

For many years, psychodynamic treatments focused exclusively on working with the therapeutic relationship, considering aspects such as transference and counter-transference. Even if this exclusiveness could be questioned and was modified in many subsequent psychodynamic developments, the work with the therapeutic relationship still offers a splendid option to reflect on and modify interaction patterns and problems in social relationships. One could argue that as long as warmth and competence are established in therapeutic relationships, there is no need for further relationship-oriented interventions. However, other experts brought attention to the fact that ruptures in the therapeutic alliance are a common phenomenon (Eubanks, Muran, & Safran, 2018), and trying to repair these ruptures can be a helpful experience not only to establish a pre-condition for a successful treatment, but also as an example of how to deal with interaction problems in everyday life (Eubanks, Muran, & Safran, 2018). A consensus between different therapists was reached on how typical ruptures during the therapeutic interaction can be categorized (Eubanks, Burckell, & Goldfried, 2018), and a portfolio on how to intervene when ruptures occur was put together (Eubanks, Muran, & Safran, 2019). Detecting and reflecting on these ruptures, and being able to use strategies from a broad portfolio how to deal with them can help further to professionalize psychotherapy.

### Reattribution and Mentalization

Reattribution takes place in all forms of successful psychological treatments. It starts with providing a new framework for understanding the clinical problem, continues with changing cognitive evaluations of one’s own feelings and behaviors, of motives of other’s behavior, and also includes reformulations of the self-concept and self-esteem. In recent years, it has been emphasized that the overall goal of all psychological treatment is to improve psychological and cognitive flexibility (Doorley et al., 2020). Psychotherapists should be sensitive and even able to trigger these reattributions. Also, models of affect regulation and its connection to psychopathology (Gross et al., 2019) emphasize the crucial role of appraisal processes. Supporting patients to be able not only to consider one explanation for problems but to choose between different views is a major step in problem solving. Cognitive therapy offers a broad spectrum of ways to deal with this topic, but it can be enriched with other approaches as well. Mentalization-based treatments (Bateman & Fonagy, 2010) also address improved perspective-taking, more variety in interpreting the motives of others and oneself, and a better understanding of emotional reactions through new appraisals.

### Working in Multi-Person Settings

For many interventions, it is necessary to work with several people together. Often, the inclusion of significant others who might play a role in maintaining the problems is necessary. But also providing group therapy (which may be more economical than individual treatments) or even working with communities belongs to the competence profile of clinical psychologists. All professional psychotherapists should be aware that the single patient always lives in a social environment with other people who interact and can either support or hamper successful changes. Therefore, working in multi-person settings is also a precondition for the broad competence profiles of psychotherapists. Systemic therapies have suggested multi-person interventions (Pinquart et al., 2016; Riedinger et al., 2017), but nearly all other major traditions of psychotherapies have developed ways to deal with it.

### Personal Competences of the Therapist

During the last decade, more emphasis has been put on the role of the persons offering psychological treatments and their personal competences. Therapist’s personality characteristics can predict parts of the treatment outcome (Norcross & Lambert, 2019). Still, there is no good and broadly accepted framework for self-reflection and self-experience and how to achieve these personality features. While the evidence for this field does not allow well-proven recommendations, there is some clinical agreement at least about one position: It is helpful if psychotherapists have the abilities they want to teach their patients (emotion regulation, communication, problem solving, self-reflection, mentalization, psychological flexibility, and even humor; Ziede & Norcross, 2020).

## An Example of Training Basic Competencies for Psychological Treatments in a University Setting

**Figure 3:**
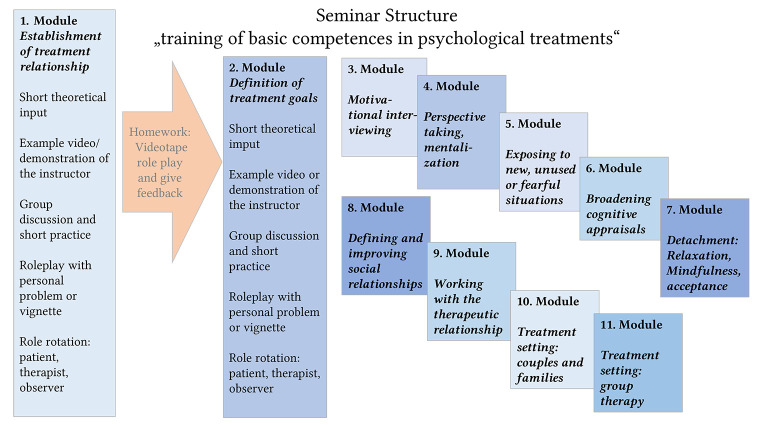
Seminar Structure

After legal regulations for providing psychological treatments in Germany changed in 2019, more practice-oriented master programs were introduced, and the University of Marburg established an example of training in basic and transtheoretical competences for psychological treatments. Hereby, the ideas of subchapter 2-4 were the basis for planning the program. In a block seminar attended by a maximum of 15 students, various modules covering basic competencies are taught (see Figure 3). Each module begins with a brief theoretical overview and repetition about one basic competence and watching an example video or a demonstration of the instructor. This is followed by a short exercise in the group and a discussion about possible difficulties and pitfalls. The main part is on role plays featuring different vignettes or personal experiences. During the first sessions, all students are required to provide a personal problem, while later, written clinical examples are the basis for the role plays. They take place in groups of three: one acting as the patient, one as the therapist, and one as the observer who provides feedback using a structured feedback form. The instructor, a licensed psychotherapist, also gives feedback. Afterwards, students rotate roles to ensure that every student has the opportunity to learn each role. Students are tasked with a homework assignment in regard to the last competence learned. They are required to create and film another role play, which is then submitted via the university's secure platform. Two randomized fellow students subsequently provide feedback on the performance and demonstrated competences of these videos.

The modules encompass a range of essential topics, including:

1. Initial establishment of a treatment relationship: In this module, students learn how to create a treatment relationship. This involves active listening, empathetic understanding, and creating a safe, non-judgmental space, allowing the patient to feel heard and supported.
2. Definition of treatment goals: Students should proactively assess both explicit treatment goals and personal objectives (life goals and values). Treatment goals should be attainable, clear, and congruent with the patient's emotional preferences. Additionally, a hierarchical approach, distinguishing between general goals (such as overall well-being and personal growth) and specific goals (like overcoming specific challenges or behaviors), allows for a comprehensive approach, addressing both immediate concerns and the broader context of the patient's life (e.g., Michalak & Holtforth, 2006).
3. Treatment motivation, motivational interviewing: The module “Motivational interviewing” employs a guiding approach to engage with patients, elicit their motivations for behavior change, and foster autonomy in decision-making; it can be learned through practicing “change talk” and “confidence talk” (e.g., Rollnick et al., 2010), and can be applied in addiction problems, but also all other ambivalence conflicts.
4. Perspective taking, mentalization: In this module, students learn to get a deeper understanding of mentalization. It refers to the capacity to understand and interpret one's own and others' thoughts, feelings, and intentions, particularly in emotionally significant interpersonal relationships, and is viewed as a learnable skill crucial for maintaining stable relationships (e.g., Bateman & Fonagy, 2010).
5. Exposing to new, unused, or fearful situations: This module provides students with specific strategies, such as expectancy violation and deepened extinction, to optimize the exposition to new situations, adding these strategies to traditional cognitive-behavioral approaches like 'fear habituation' and 'belief disconfirmation' (e.g., Craske et al., 2014).
6. Broadening cognitive appraisals: In this module, students learn how to expand the patients´ perspective and how to consider alternative interpretations of situations to gain more balanced perspectives and constructive thinking patterns. Developing psychological flexibility is a major goal.
7. Detachment: This module trains the integration of detachment, encompassing relaxation, mindfulness, and acceptance, as an important aspect of psychological treatments with a focus on emotion regulation (Shapiro et al., 2006). Techniques such as progressive muscle relaxation, mindfulness meditation, and self-compassion practices are employed to help patients cultivate detachment from thoughts and emotions (Wells, 2005).
8. Defining and improving social relationships: In this module, understanding and improving social behavior is trained with the help of the Interpersonal Circumplex model (Kiesler, 1983). This framework visualizes interpersonal behavior along two axes: agency (ranging from dominance to submissiveness) and communion (ranging from friendliness to hostility), creating a circular space. This model categorizes behavior into eight segments, providing a comprehensive representation of an individual's interpersonal profile and serving as a valuable tool for understanding psychopathology within social contexts (Guhn et al., 2019).
9. Working with the therapeutic relationship; complimentary relationship expectations; ruptures and repair: In this module, the students learn the concept of complementary therapeutic relationship, in which therapists should offer each patient a customized relationship tailored to their most significant goals, as determined through plan analysis and case conceptualizations. This approach suggests that a therapist's behavior should align with and complement the patient's needs and objectives in therapy (e.g., Caspar et al., 2005). Moreover, students train to recognize and effectively address ruptures in therapy (e.g., Eubanks et al., 2019).
10. Treatment setting - couples and families: This module stresses the importance of integrating the partners and children into the psychological treatments. It offers strategies for enhancing positive interactions and components for communication, and it also trains the therapist in multi-perspectivity and impartiality. Additionally, it outlines therapeutic approaches for various psychological disorders within the framework of couple and family therapy (e.g., Hahlweg & Baucom, 2008).
11. Treatment setting - group interventions: In this module, students learn to conduct different types of group therapy (e.g., psychoeducational, disorder-specific, individual case-oriented). The main focus of the module is on individual case-oriented group therapy addressing the specific psychological issues or predetermined theme of a protagonist. The selection of the topic is tailored to the individual's personal situation and life history, with the assumption that most group participants may have similar problems or life situations (Sipos & Schweiger, 2019). The goal is to work on the individual situation of the protagonist, while other group members serve as sources of information, experiences, feedback, and practice partners for role-playing exercises. All of the students get different vignettes about their role as trainer or participant, and large role plays follow (see Supplementary Materials, Table S2 for an example vignette).

**Figure 4:**
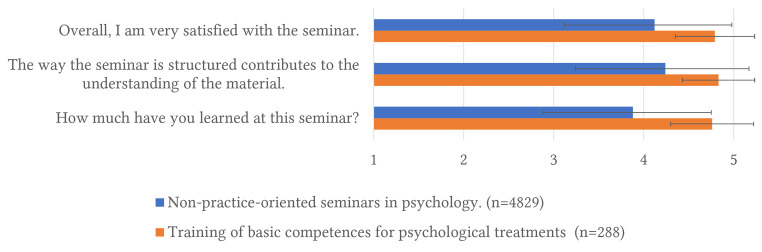
Teaching Evaluation of the Training of Basic Competences for Psychological Treatments *Note.* Legend: The items are rated on a five-point response scale ranging from 'strongly disagree' = 1 to 'strongly agree' = 5, or 'very little' = 1 to 'very much' = 5.

The described seminar is evaluated regularly, using standardized questions that are similar in most German universities. The evaluations consistently show that students rate the quality of the seminar very high. Figure 4 shows the students' assessment regarding the three most relevant items for evaluating seminar quality (satisfaction, understanding of the material, increase of learned content). The results of the training of basic competences for psychological treatments include 15 teaching evaluations (2012-2023) from six different instructors. For comparison, *n* = 4,829 teaching evaluations from non-practical events in psychology from the same years are depicted.

Taken together, the seminar integrates theoretical knowledge with hands-on practice. It empowers students to apply basic competences in practical settings and receive feedback from both instructors and peers. Such active learning strategies equip students for real-world applications of their psychological treatment skills.

### Shortcomings of This Approach

The competence-based training approach in psychotherapy, while valuable in many respects, is not without its limitations. It is important to acknowledge that this approach is not intended to replace comprehensive postgraduate trainings in psychotherapy, but it is meant to offer an alternative to current trainings in particular as a starting approach early in the career, e.g., offering a “common trunk” before specialization takes place. Here are some of the key shortcomings associated with this approach:

Lack of Disorder-Specific Approaches: One significant limitation is its generalist nature. It may not sufficiently cater to the unique needs and nuances of specific psychological disorders and problems. Tailoring interventions to address specific conditions like depression, anxiety, or trauma requires additional training and expertise.

Incomplete Coverage of Competences: While the competence-based approach covers important therapeutic skills, it may not encompass the full spectrum of competences that could be beneficial in psychotherapy, and it will always represent a selection. Factors such as cultural sensitivity, advanced assessment techniques, or specialized interventions for severe psychopathologies might not receive adequate attention. The complexities of transference and countertransference, which are crucial aspects of the therapeutic relationship, may not be fully addressed in a competence-based framework, similar as some other specific approaches (such as schema therapy or specialized exposures). Emotion regulation training could be strengthened compared to this proposal. There are limitations in a transtheoretical approach to integrating highly specialized abilities from all different approaches. However, we want to understand our approach as a dynamic model that invites modifications, adaptations, and improvements and also allows variations. In contrast to being bound to one single approach, a strength of our approach is that it can be flexibly adapted to improve identified shortcomings and integrate new evidence-based acknowledgements stemming from different fields.

In summary, while the competence-based approach provides a valuable foundation for psychotherapists, it should be viewed as a starting point rather than a final comprehensive training in itself. Supplementing this approach with specialized knowledge, disorder-specific techniques, and a nuanced understanding of complex therapeutic dynamics is essential for providing high-quality, tailored care to clients with diverse needs.

## Concluding Remarks

Many early career clinicians using psychological treatments receive training that focuses on one of the traditional or current frameworks, such as psychodynamic, CBT, or ACT. Focusing on one of these approaches, often accompanied by developing a strong identification for it, typically neglects other experiences, new developments in other contexts, and/or basic findings on disorders or treatment mechanisms. Overcoming these limitations requires a transtheoretical approach for case conceptualization and treatment. This can create a platform for a true academic and scientific field of psychological treatment. We provide such transtheoretical frameworks for case conceptualization, and we suggest a competence-based framework for training early career clinicians in how to use psychological treatments. These concepts should not be understood as fixed or new truth, but as a flexible framework that can be continuously adapted according to new scientific or practical experiences and local needs. We established a basic training of competences for upcoming psychotherapists that integrated treatment approaches of different theoretical orientations. Students’ satisfaction was very high, and negative aspects (e.g., being confused; not being able to integrate the different approaches to an overall understanding) were not observed. Broadening the science of psychological treatments to a transtheoretical approach helps to overcome artificial differences and improves the integration of our knowledge and experiences into an overall transtheoretical framework.

## Supplementary Materials

The Supplementary Materials include the following items (for access, see Rief et al., 2024):

- The first supplemental material outlines a framework detailing essential competencies for psychological interventions with individuals facing persistent physical health conditions. These include professional stance, values, and assumptions; core knowledge about the illness; a good assessment and planning ability; generic therapeutic competences such as the ability to foster and maintain a good therapeutic alliance; knowledge about specific interventions; and meta-competences.
- The second supplement provides an excerpt of a competence list, as outlined by the EACLIPT Task Force on "Competences of Clinical Psychologists" in 2019. Meta-competences for clinical psychologists encompass proficiency in providing interventions aligned with treatment aims and scientific knowledge. Moreover, meta-competences include the ability to motivate patients, explain interventions to stakeholders, demonstrate perspective-taking and empathy, regulate their own emotions, and address treatment and therapeutic relationship issues.
- The third supplemental material introduces a case vignette illustrating therapeutic objectives aimed at addressing relationship ruptures and understanding patient motives to enhance therapeutic engagement and flexibility. This case features a patient with an affective disorder and can be used in the training of clinical psychologists.

RiefW.
WilhelmM.
BleichhardtG.
StraussB.
FrostholmL.
von BlanckenburgP.
 (2024). Supplementary materials to "Competence-based trainings for psychological treatments – A transtheoretical perspective"
[Additional information]. PsychOpen. 10.23668/psycharchives.14202
PMC1130393039118657
